# An assessment of the genomic structural variation landscape in Sub-Saharan African populations

**DOI:** 10.21203/rs.3.rs-4485126/v1

**Published:** 2024-07-08

**Authors:** Emma Wiener, Laura Cottino, Gerrit Botha, Oscar Nyangiri, Harry Noyes, Annette McLeod, David Jakubosky, Clement Adebamowo, Phillip Awadalla, Guida Landouré, Mogomotsi Matshaba, Enock Matovu, Michèle Ramsay, Gustave Simo, Martin Simuunza, Caroline Tiemessen, Ambroise Wonkam, Venesa Sahibdeen, Amanda Krause, Zané Lombard, Scott Hazelhurst, as members of the H3Africa Consortium

**Affiliations:** 1 Sydney Brenner Institute for Molecular Bioscience, University of the Witwatersrand, Johannesburg, South Africa.; 2 Division of Human Genetics, National Health Laboratory Service and School of Pathology, Faculty of Health Sciences, University of the Witwatersrand, Johannesburg, South Africa.; 3 Computational Biology Unit, University of Cape Town, Cape Town, South Africa.; 4 College of Veterinary Medicine, Animal Resources and Biosecurity Makerere University, Kampala, Uganda.; 5 Centre for Genomic Research, University of Liverpool, Liverpool, United Kingdom.; 6 Institute of Biodiversity, Animal Health and Comparative Medicine, University of Glasgow, Glasgow, United Kingdom.; 7 Department of Biomedical Informatics, University of California, San Diego, United States of America.; 8 Institute of Genomic Medicine, University of California, San Diego, United States of America.; 9 Department of Epidemiology and Public Health and Greenebaum Comprehensive Cancer Center University of Maryland School of Medicine, Baltimore, United States of America.; 10 Ontario Institute for Cancer Research, Toronto, Canada.; 11 Department of Molecular Genetics, University of Toronto, Toronto, Canada.; 12 Dalla Lana School of Public Health, University of Toronto, Toronto, Canada.; 13 Faculty of Medicine and Odontostomatology University of Sciences, Techniques and Technology of Bamako, Bamako Mali.; 14 Neurology Department Point ”G” University Hospital, Bamako, Mali.; 15 Botswana-Baylor Children’s Clinical Center of Excellence, Gaborone, Botswana.; 16 Baylor College of Medicine, Houston, United States.; 17 Molecular Parasitology and Entomology Unit, Department of Biochemistry University of Dschang, Dschang, Cameroon.; 18 Department of Disease Control, School of Veterinary Medicine University of Zambia, Lusaka, Zambia.; 19 Centre for HIV and STIs, National Institute for Communicable Diseases, National Health Laboratory Services and Faculty of Health Sciences University of the Witwatersrand, Johannesburg, South Africa.; 20 McKusick-Nathans Institute and Department of Genetic Medicine, Johns Hopkins University School of Medicine, Baltimore, United States of America.; 21 Division of Human Genetics, Faculty of Health Sciences, University of Cape Town, Cape Town, South Africa.; 22 School of Electrical & Information Engineering, University of the Witwatersrand, Johannesburg, South Africa.

**Keywords:** Structural variants, African diversity, copy number variants, genomic variation

## Abstract

Structural variants are responsible for a large part of genomic variation between individuals and play a role in both common and rare diseases. Databases cataloguing structural variants notably do not represent the full spectrum of global diversity, particularly missing information from most African populations. To address this representation gap, we analysed 1,091 high-coverage African genomes, 545 of which are public data sets, and 546 which have been analysed for structural variants for the first time. Variants were called using five different tools and datasets merged and jointly called using SURVIVOR. We identified 67,795 structural variants throughout the genome, with 10,421 genes having at least one variant. Using a conservative overlap in merged data, 6,414 of the structural variants (9.5%) are novel compared to the Database of Genomic Variants. This study contributes to knowledge of the landscape of structural variant diversity in Africa and presents a reliable dataset for potential applications in population genetics and health-related research.

## 1 Introduction

Structural variants (SVs) are rearrangements of genomic content that are typically greater than 50bp in size, including deletions, duplications, insertions, inversions, translocations, and more complex rearrangements. Copy number variants (CNVs) are a subset of SVs that result in a change in the number of copies of a genomic region. SVs are responsible for a large part of genomic variation between individuals [1, 2, 3] but also play a role in disease, having been implicated in both common diseases such as cancer [4], and rare diseases, including many neurodevelopmental disorders (NDDs) [5, 6]. Unfortunately, the full characterisation of SVs has been slowed by the complexity and technical challenges of SV discovery. These challenges have resulted in a delay in publicly available high-quality SV databases from large population studies, which in turn has limited our understanding of the precise role of SV in human variation and disease [1].

Understanding the variation of the population informs research on genetic disorders. Early studies to map human SVs and understand their role in human diseases were performed using microarray genotyping technology [7, 8, 9]. These studies began to uncover the role of SVs in disease [10], but did not have the break-point accuracy and high resolution required for the discovery of small SVs [11]. With the increasing number of human genomes sequenced using next-generation sequencing (NGS) approaches, methods to detect SVs from these data were developed. Studies such as the 1000 Genomes Project[11] and the Genome Aggregation Database (gnomAD) [2] have produced landmark SV datasets that greatly contributed to understanding the global distribution of SVs.

Current SV reference databases are biased towards European populations and contain few African genomes, especially from continental Africans, thus representing only a subset of global genetic diversity [12, 13]. The most comprehensive African study to date is the study of 232 individuals using medium-coverage (10x) data [14]. The lack of knowledge of African genomes hampers the goal of access to genomic medicine initiatives for all global populations. As such, high-quality data sets of baseline variation in African populations will enrich our knowledge of the human genome [13] and help to interpret potentially pathogenic variants in the study of genetic diseases [15, 16]. Through our comprehensive dataset, we build on prior work to explore the breadth of SV diversity in Africa and establish a reliable reference of African SVs for potential applications in population genetics and health-related research.

## 2 Results

**Fig. 1:**
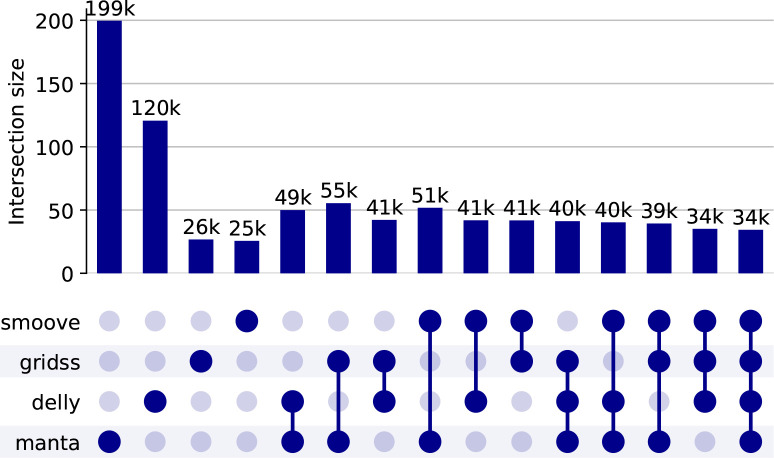
Overview of the calls found by each SV caller, and combining them using SURVIVOR. See the methods section for details of choices. Note that in this graph we abuse the standard UpsetPlot representation – for example, 49,000 variants are detected by both Delly and Manta which may or may not be detected by other algorithms (in the standard UpsetPlot the implication would be that they are *not* detected by other approaches). Note also the anomalous results for gridss and smoove is caused by the fact that joint calling is not done by these tools and so estimating the actual number of SVs is very difficult.

We ran five different SV tools – Manta (1.6.0–3) [17], Delly (1.1.6) [18], GRIDSS2 (v2.13,2)[19], smoove (v0.2.8) [20], and CNVPytor (v1.2.1) [21]) on the 1,091 high coverage WGS. Figure 1 shows an overview of the calls found by each tool and combinations of the tools. As discussed in the Methods, we use as our consensus set any SVs found by at least three tools, and this consensus set is discussed in the rest of the section.

### 2.1 Overview of calls of length ≤10,000bp

We first reviewed the calls that are ≤10,000bp in length and are so validated by three of the callers. There are 67,795 SVs ≤10,000bp in length, 36,981 in intergenic regions, and 30,814 in gene regions (including the untranslated regions). A total of 10,421 genes contained SVs. Categorised by type of structural variant, the number of variants are shown below in Table 1. However, note that due to the differences in the way that different callers and SURVIVOR call and handle variants that this is only a rough guide, determined as much by the power of the tools as the actual range of variants.

As is expected most SVs occur at low frequency (22,551 singletons), 38% have a frequency less than 1%, however, 17% occur at greater than 10% frequency. More variants occured at higher frequencies compared to the gnomAD SV study where only 10% of variants had frequencies >1%. Table 2 below gives an overview of frequency.

#### Novel SVs discovered

Comparison with the Database of Genomic Variants (DGV)[22] was performed using a cut-off of a reciprocal overlap of 70/90/95% and showed 6,414/9,483/11,673 new SVs respectively. Using a conservative 90% reciprocal overlap, 14% (9,483) of the dataset were novel African SVs, not previously described in DGV. This large number of unique variants highlights that African individuals harbour a substantial amount of previously undescribed structural variation. The population structure analysis described below further shows that not only do African individuals have unique variation overall, but many of these variants differ between African regions in sub-Saharan Africa.

#### Length spectrum

**Fig. 2:**
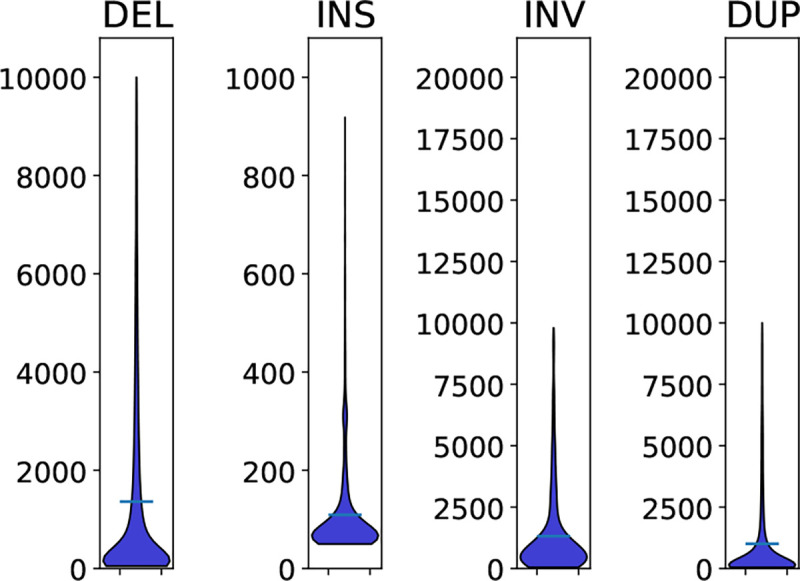
Violin plot of distribution of lengths

Showing the spectrum of lengths is complex. Multiple samples may have SVs that *are* slightly different or that the SV caller *reports* as slightly different. Furthermore, different tools estimate SVs of different lengths. Figure 2 and Table 3 show the distribution of lengths of SV calls and as expected we see the majority of SVs are small with 70% being less than 1,000bp.

#### Per-individual variants.

**Fig. 3:**
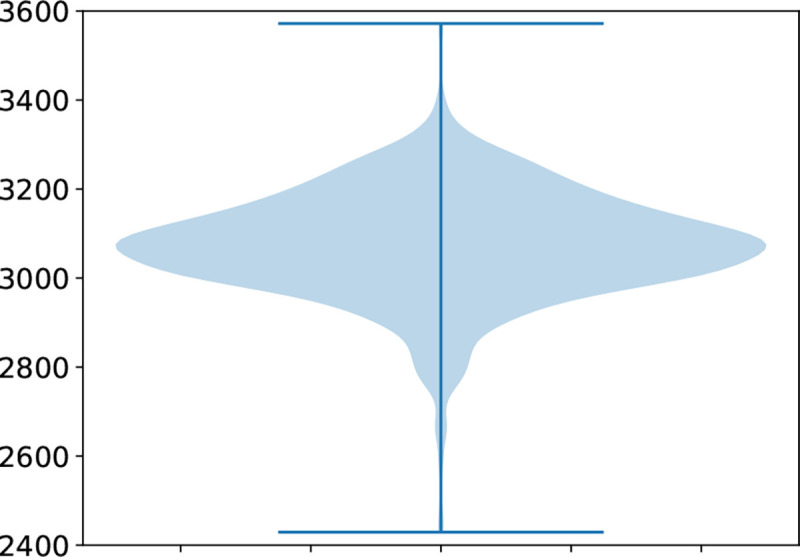
Overview of number of variants per individual.

On average there were 3073 variants per individual (range [2429–3572], standard deviation = 123). Figure 3 shows an overview.

### 2.2 Annotation and impact of the calls ≤ 10,000bp in length

Annotation by AnnotSV[23] shows that the majority of the SVs are likely benign, as they do not include genes previously implicated in disease, but that there are a number that may be deleterious. Table 4 summarises predictions made regarding American College of Medical Genetics (ACMG) classification. Table 5 shows the 15 variants predicted to be pathogenic. Of the 15 variants, nine of them are present in population SV databases and almost all these were found uniquely in African individuals in previous studies. With our large African cohort we can add additional frequency data to these variants. The deletion involving *PTCH2* present in one individual in this cohort, is an interesting finding. It is accepted that *PTCH2* a homolog of *PTCH1* may also cause nevoid basal cell carcinoma syndrome, and one such variant is reported in ClinVar as a pathogenic, one-star classified variant (VCV000059967.1). However this association is under dispute as reports of healthy indiviudals with these variants have been published[24, 25]. This deletion involving *PTCH2* may be another such case. The remaining five variants have not been previously reported in population databases and are all singletons except the deletion involving most of *GNPTG* and the beginning of *UNKL*. This variant was found in three individuals in the cohort and partially overlaps a variant reported in ClinVar as pathogenic, but is not present in any population databases. Most SNVs in *GNPTG* are associated with mucolipidosis III an autosomal recessive condition. With a deletion of most of the *GNPTG* gene these individuals are likely heterozygous carriers for a novel pathogenic African variant.

Table 6 summarises how structural variants overlap with genomic regions. Compared to population SNVs an unexpectedly high proportion of variants falls within the range of a gene. Almost all of these are however intronic variants. In their paper Collins et al. [2] showed that selection against variants is highly correlated to size of the SV and the functional impact. They calculated an adjusted proportion of singleton score to compare selection against different SV types, taking into account genic location. Intronic variants like other non-coding variants score close to null, second to intergenic variants, indicating little selection against them.

### 2.3 Population Structure

**Fig. 4:**
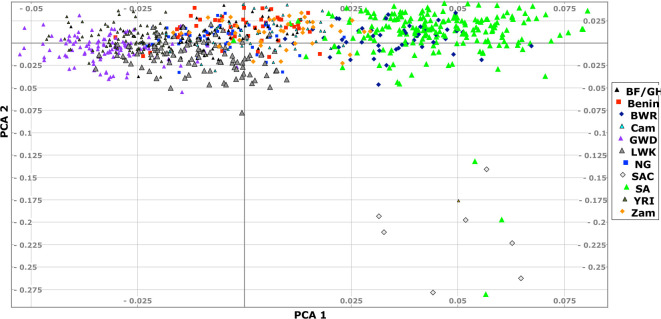
Principal component analysis of the SVs less than 10,000 in length. Some populations are omitted for clarity. Key: ACB – 1000G African Caribbean in Barbados; Benin – H3Africa; BF/GH – H3Africa Burkina Faso and Ghana; BWR – H3Africa Botswana; CAM – H3Afica Cameroon; GWD – 1000G Gambian Western Districts; LWK – 1000H Luhya from Kenya; MSL – 1000G Mandinka Sierra Leone; NG – H3Africa Berom from Nigeria; SA – CBRL, H3Africa, Bantu-speakers from SAHGP South Africa; SAC – SA Coloureds in SAHGP; YRI — 1000G Yoruba; Zam – H3Africa Zambians.

As expected, SVs differ across the continent. A principal component analysis (PCA) using PLINK2 [26] shows that populations can be differentiated with significant variation from south to centre and east to west – Figure 4 shows the PCA. Although the structure is not as defined (first two eigenvalues are 4.3 and 3.7) as for the SNVs shown in Figure A1 (first two eigenvalues 57 and 13), the structure is clearly visible.

### 2.4 Calls that are >10,000bp in length

This class of calls is reported separately because a different method was used for combining results, and the consequences of false positives with very long SV regions in annotation are more serious. Preliminary investigation showed that there were many SV regions much greater than 10,000bp, some with very strong evidence. While some of these calls are indicative of some structural rearrangement it is highly unlikely that they are as long as the callers indicate. Therefore, a different approach was taken.

First, we required that one of the depth-based approaches and another method validate the call. Second, we only analysed calls that are less than 200kbp long, since incorrect longer calls are likely to overlap many genes and pollute downstream analysis. We have however, made BED files available for all calls.

**Fig. 5:**
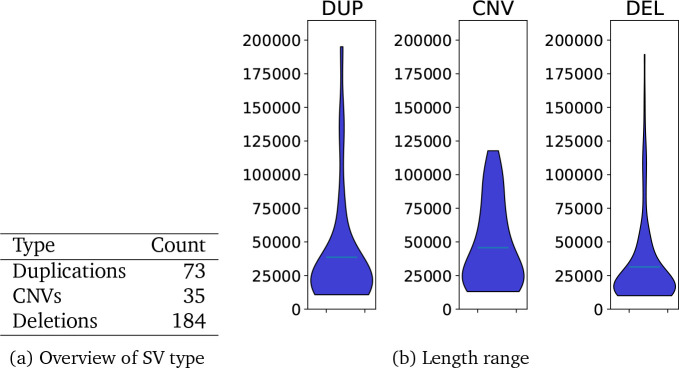
Overview of SV regions between 10,000bp and 200,000bp in length

There were 291 SV regions in total. More detail can be found in Figure 5. A disproportionate number of these longer SV regions occur on chromosome X (158). Although with a few exceptions, the SV regions do not occur in the pseudoautosomal regions, this may be due to failure of the methods to properly account for chromosome X. Of the SV regions that do not appear on chromosome X, 41 completely covered a gene. The bed file of all the longer SV regions is available as supplementary data.

## 3 Discussion

Structural variation research worldwide has been slowed by the fact that SVs are highly varied in nature and technically challenging to identify with high confidence. Given the universal under-representation of African genomic data in the public domain, baseline population SVs have been understudied in African populations. We analysed 1,091 high-coverage African genomes to address this representation gap.

Applying an ensemble call approach, we identified 67,795 SVs throughout the genome, with 10,421 genes having at least one SV. Categorising SVs by subtype is complex and biased by the capabilities of SV-detection algorithms, but with that caveat, 75% of the variants were deletions, 19% duplications, 4% insertions, 2% inversions, and 12 translocations.

There was considerable variation in SV size, with an expected majority of variants being small. About 41% are between 50–200bp, 25% between 200–800bp, 20% between 800–3,200bp, and 13% between 3,200–12,800bp. No SVs longer than that met our criteria for SV-detection although individual methods were able to detect longer SVs. These proportions support the premise that size is one of the greatest factors affecting selection against SVs [2]. Again, the numbers we report are biased by the ability of different algorithms to detect different length SVs, but they do align with expected proportions.

When looking at the frequency of variants detected we see a higher number of common variants than expected. 17% occur at greater than 10% frequency. More variants occured at higher frequencies compared to the gnomAD SV study where only 10% of variants had frequencies >1%. Given that African ancestry individuals have been known to have a greater number of SVs, this greater number of common variants may be a feature of the African SV landscape.

On average, the individuals had ≈ 3000 SVs; very few individuals varied more than ≈10% from this number. This number is lower than that reported in the gnomAD-SV study for African ancestry genomes. This could be due to the smaller size range included in the analysis. In this study, no variants larger than 12,800bp were included, where in the gnomAD-SV study they resolved variants from 50bp–10Mb. They also utilised different tools which will affect variants detected.

At first glance a surprising proportion of SVs were near genes – 42% occurred in introns, 4% affected coding regions with 53% being intergenic. As mentioned earlier, when considering the location of SVs their functional impact and size have to be taken into account. If SVs are small enough to only involve non-coding introns they are unlikely to be highly selected against and therefoore will be found at relatively high proportions. Collins et al. [2] utilised normalised singleton proportions to estimate these selection pressures and indicate that intronic variants are almost as unlikely to be selected against as intergenic regions.

With respect to predicted functionality, 68% of SVs have uncertain impact according to ACMG classification, 25% are predicted as benign, 0.2% *likely pathogenic* and only 15 are predicted as pathogenic. Given that the participants were all adults recruited as part of population cross-sectional or infectious disease studies, the relatively small number of pathogenic predictions is expected. Some of the pathogenic and likely pathogenic variants, identified here occur in a heterozygous form, and are therefore not disease causing. However, recording the frequency of these variants more accuratley remain important, as it gives a clearer indication of the mutation spectrum for the respective diseases that these variants in homozygous form contribute towards.

Using a conservative overlap, 6,414 of the 67,795 variants (9.5%) are new SVs compared to the DGV. One of the aims of our study was to add to the catalogue of known SVs in current references databases, as such databases have an underrepresentation of African genomic data. This reduces their usefulness to researchers studying individuals of African ancestry. High quality datasets of baseline population African variation, and analysis thereof, are of critical importance as they aid in the interpretation of potentially pathogenic variants in the study of genetic diseases in Africa.

In this study we encountered the multiple reasons why SV research has lagged behind SNV research. Technical and analysis challenges complicate accurate calling of SVs using short read sequencing technologies. Short read data (e.g., 150bp even if paired end) is not ideal for studying SVs. There are several signals that can be used – e.g., read depth, different parts of reads aligning to different places on a genome, unexpected difference where two paired-end reads align to the reference – but variants of increased length are more difficult to detect accurately. While moves to pan-genomes and reference graph representations may partially assist, there is no doubt that high quality long-read sequencing data is much better suited for SV detection, especially in understudied populations [27].n

Different SV-detection algorithms have different advantages as different approaches are employed. Given what is known of the relatively high error-rate (false positives and –negatives), ensemble calling has become common. How to combine callers is not straight-forward as different tools work better for different SV lengths. For example, algorithms that use read-depth as a criterion for CNVs are suitable for detecting variants several thousand bp in length, while other tools that use read content work well for SVs that are 50–70bp in length. Thus simply combining two such tools by requiring that *both* tools must detect a variant is bound to miss the variant while allowing either tool to detect the variant improves sensitivity but does not impact on false positive rate. We took a conservative strategy – as discussed in Section 5.3.3. Even SV calls with strong evidence using two different tools may lead to incorrect conclusions. For variants less than 1,000bp we requiried that three of four tools must detect an SV. This strict approach meant that in some cases variants called by fewer than three tools in an individual did not proceed to the final merging step, resulting in lower variant frequencies for known common variants.

Each SV-caller is very computationally intensive. Some tools only accept BAM files with individual samples in the range 70–90GB in size. Tools often produce intermediate data files that require significant extra disk space. All algorithms require processing the BAM files once and some twice, and the detection algorithms are complex, so this project has used several hundred thousand CPU hours and hundreds of TB of data. Exploring different approaches and parameters with a significant sample size is therefore a computational challenge.These challenges have significantly hindered the discovery of the African SV landscape and therefore we are making this SV dataset available to the global research community as an important resource to enhance the discovery of functional impact of SVs in the genome.

## 4 Conclusion

This paper introducing a database of SVs in African populations, contributes to knowledge of the landscape of human diversity with a focus on Africa. We have identified a large number of SVs previously undiscovered in other populations that, together with existing data sets, will assist future work into understanding the functional impact of genomic variation and will become a valuable resource for researchers and clinicians studying individuals with African ancestry.

## 5 Methods

### 5.1 Data sets

We used high coverage (> 30×), short read, WGS data from 1,091 individuals obtained from various sources, including the Human Heredity and Health in Africa (H3Africa) consortium [28], the 1000 Genomes Project [11], Simons Genome Diversity Project [29], the South African Human Genome Programme [30] and other unpublished African resources. Table 7 gives an overview of the populations represented in this study, and is further described in Appendix A. Figure A1 shows the regional distribution of the included datasets.

### 5.2 Data alignment to reference

The 1000 Genome Project VCFs were created using the protocol described in [31]. We applied the same protocol to all other samples. Briefly, reads were aligned with build GRCh38 using BWA (v.0.17- r1188) [32]. Finally, we performed alignment duplicate marking with GATK MarkDuplicates (v4.1.3) and quality score recalibration with GATK Quality Score Recalibrator (v4.1.3) to improve the quality of the alignment BAM file.

### 5.3 Variant calling

We used several SV and CNV calling tools and produced a consensus dataset, with the Nextflow scripts used accessible here: (https://github.com/shaze/h3acnvalls). We first describe the methodological approach for consensus calling and then detail the calling and merging steps.

#### 5.3.1 Methodological approach

Producing a dataset from many samples using multiple tools requires merging both across samples and tools. Ideally, calling across samples would be done jointly to improve quality of calls and produce cleaner datasets. However, not all calling tools are capable of joint calling, especially on larger datasets. There are three complications in producing a merged dataset. First, SVs and CNVs vary more than single nucleotide variants (SNVs) – both in the type of variant and in the amount of the genome affected by the variant. For example, two samples may have insertions in overlapping regions of the genome that may not be identical. Whether these should be recorded as one or as two SV regions depends in principle on how similar the insertions are (do they differ in content very slightly and so likely are products of the same evolutionary event, or very differently and to what degree do the called regions overlap). Second, the difficulties in calling and limitations of calling tools and random variation in the sequencing of different samples (especially with short reads) means that even if two samples have exactly the same variant, a caller may report them slightly differently. Third, SVs and CNVs are complex, and the various tools used for merging have limits and are not always regularly updated. A simple case is that one caller may call a duplication (DUP) and another an insertion (INS), CNV, or breakpoint (BND) with minor differences in the actual sequencing. More complicated variant annotations (BND, translocation [TRA], inter-chromosomal translocation [CTX], intra-chromosomal translocation [ITX]) are challenging because a caller may produce an annotation that the other does not recognise, and the rules for how combinations are performed may be hidden in code. These considerations impact the decisions made in the protocols described below.

Furthermore, even tools capable of “joint calling” produce outputs that require postprocessing. Joint calling is generally done by first running the tool independently on each sample, merging all the sites where SVs were detected across all samples, and then regenotyping the individual BAMs on the merged site list. For example, a tool detects a duplication in sample *A* on chromosome 1 at location 10,000 to 10,300, and also detects a duplication in sample *B* on chromosome 1 at location 10,010 to 10,298. In reality, these two SVs are likely the same SV, but the tools treat them as independent SVs. In the re-genotyping step both sites are checked for all samples – there is a high-probability that both SVs will be called on both samples. On sample *A*, the first deletion may have a better quality score than the second deletion, but the second deletion will also have a very good score. To remedy this, we use ‘stitching’ as described below.

#### 5.3.2 Calling

**Fig. 6:**
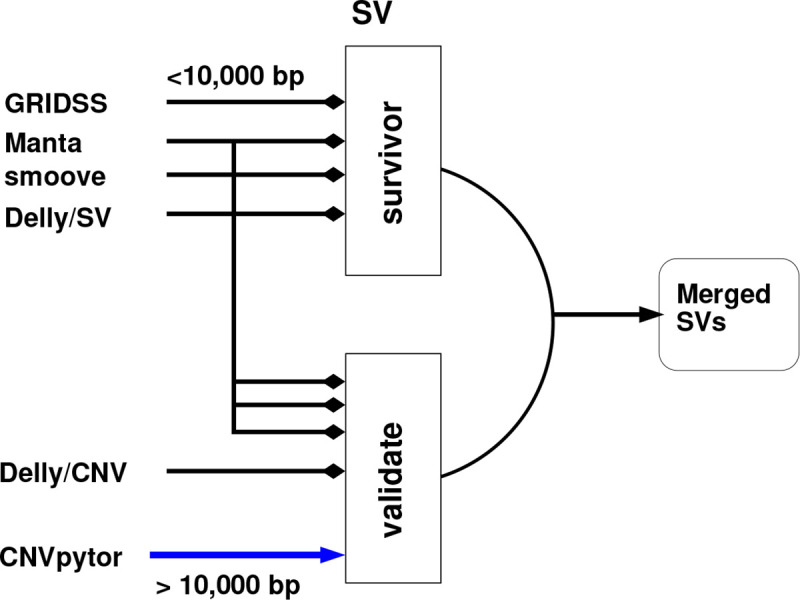
Overview of merging process: Variants that are less than 10,000bp in length must be supported by at least 3 tools. Variants greater than 10,000 bp must be supported by CNVPytor, a depth-based method, and one other tool.

Many tools have been developed to call SVs from short-read WGS data. As each tool has its own limitations and advantages, the use of a variety of SV/CNV-calling algorithms can generate a more comprehensive dataset. As such, SV/CNV calling was performed using several different tools. We then combined the results from the different tools and performed joint calling using SURVIVOR (Figure 6).

Five tools were used because they make use of different combinations of approaches for calling SVs and CNVs – Manta (1.6.0–3) [17], Delly (1.1.6) [18], GRIDSS2 (v2.13,2)[19], smoove (v0.2.8) [20], and CNVPytor (v1.2.1) [21]). Delly has two modes of operation: SV detection and CNV detection (which will be referred to as Delly/SV and Delly/CNV, respectively). CNVPytor detects CNVs while the other tools detect SVs.

GRIDSS2 extracts reads that are potentially indicative of SVs from BAM files (split reads, discordantly aligned reads, soft-clipped reads and reads anchored only on side of the read). A positional de Bruijn graph is constructed and analysed to identify the SVs. We followed the recommended approach for calling germline SVs except that (a) we did not do joint calling (since this is done with SURVIVOR and the GRIDSS developers suggest that joint calling of hundreds of samples is computationally infeasible) or exclude any regions; and (b) we did not exclude the ENCODE Data Analysis Center blacklist of problematic regions in the genome [33] since we did this jointly across all data calls. We used the GRIDSS2-recommended post-analysis of break-end (BND) variants to determine simple variant types like INS, DEL and DUP and used Truvari [34] to merge variants that were close to each other.

Delly performs SV calling using a combination of split-read and read-pair information. Briefly, for both Delly algorithms individually, the process is: (a) each sample is separately called; (b) all sites found across all individuals are merged; (b) each sample is called again using the common sites to improve accuracy; (c) the resultant calls are merged and (d) filtered using Delly’s SV filter, and then (e) split into individual files for later processing. Delly has a separate read-depth CNV calling method –– the results of the SV caller are used to refine the start and end points of the variant. We used the recommended protocol for detecting germline SVs with Delly: SVs are called using the BAM files of individual samples, and the call sites between samples are merged. The individual BAM files are regenotyped using the combined merged list. For CNV calling, each BAM is called independently using the SV calls from that sample as auxiliary information, the sites are merged and then each BAM is re-genotyped on the combined site list.

Manta [17] uses break-end information as its primary SV detection mechanism. SVs are detected per sample; there is no type of joint calling. The first phase of the algorithm builds a break-end graph using the aligned reads. Once the break-end graph is created, reads aligning to each break are analysed and assembled, and evidence for different types of SVs is evaluated.

Smoove [20] is a wrapper and workflow for LUMPY [35]. LUMPY uses read-pair, split-read, and read-depth information to make probabilistic calls of SVs. Joint calling is supported. We followed the recommended workflow of (a) calling SVs on each BAM separately; (b) merging all called sites; (c) re-genotyping each BAM using the merged site list; and (d) combining (“smoove paste”). We then annotated and filtered out poor quality calls.

##### Counts of variants for individual tools

For tools that support joint calling, this can be computed directly from the joint output. For GRIDSS and Manta, for which joint calling is not supported, after removing poor quality calls the method proposed by Truvari [34] was followed: the VCF files were merged using bcftools 1.15 [36]. The resulting file was split into deletions and non-deletions and then Truvari was used on each file to merge SVs from different samples that overlap. The resulting files were then merged and sorted. The parameters can be found on our Nextflow scripts.

CNVPytor is primarily a depth-based CNV caller. We computed read-depth and called CNVs independently on each BAM and using a window size of 1,000 bp. Although, CNVPytor’s merge in principle supports some sort of joint calling we were unable to produce useful output. The results of CNVPytor’s calling is in windows of size 1,000 bp resulting in imprecise variant boundaries, which impacted how we combined results.

#### 5.3.3 Merging results

When combining the subsequent variant lists from each tool, we only used variants that passed quality control according to each tool specification. Although there is an argument for including variants with weaker evidence when doing ensemble calling, we chose to be strict. After preliminary experimentation, we used the hybrid approach for ensemble calling, depending on variant length, shown in Figure 6. After preliminary analysis, we filtered out insertions greater than 1,000bp, deletions greater than 10,000bp, inversions greater than 20,000bp and duplications greater than 20,000bp.

For variants that are ≤ 10,000bp, we use SURVIVOR [37] to select variants that were supported by any three of the following tools: Manta, Delly/SV, GRIDSS and smoove. There are relatively few variants larger than this cutoff point. However, we excluded longer variants: manual inspection of examples showed very high-quality evidence for some sort of SV, but the lengths were implausible and in some cases caused thousands of genes to be implicated in an SV. The risk of false positives or misinterpretation outweighs the value of such calls.

Different support values *s* (2, 3, 4) were tested, which means how many tools called the same variant. For each sample, SURVIVOR was used to combine the results of the four different tools so that at least *s* of the tools supported the call for a variant. SURVIVOR then combines the per-sample results to produce an overall jointly called file. Finally, the ENCODE DAC regions are excluded. For the main analyses, a variant must be called by at least three tool (*s* = 3) was used. Preliminary analysis showed that different tools annotate variants differently (e.g., INS, DUP, BND). As such, we used SURVIVOR’s mode of ignoring the variant type to decide to call a consensus.

For longer variants (>10,000bp), we required that they be called by CNVPytor and at least one of the other callers. This means that such calls are supported by both read-depth and sequence content. As CNVPytor has very imprecise calls, SURVIVOR was inappropriate, and we used our own script validate call. Therefore, we took all variants longer than 10,000 bp found by Manta, Delly/SV, Delly/CNV, and smoove and selected those that had at least a 90% overlap with a call from CNVPytor. GRIDSS calls were all shorter than 10,000bp.

### 5.4 Variant annotation

Annotation of the SVs ≤ 10,000 bp was performed using AnnotSV [23]. AnnotSV reports whether SV regionss are intergenic, coding, or in another genic or non-genic region. It also predicts variant classification according to the ACMG guidelines [38]. We merged SV regions that overlapped by at least 70%, covered the same genes, and had the predicted SV type (either identical or both [DUP, CNV] or [DEL, CNV]).For larger SV regions, AnnotSV was unable to successfully complete the annotation.

### 5.5 Comparison to Established Databases

To determine which variants in our data set are novel, we compared them with the DGV [22], version (GRCh38 hg38 variants 2020-02-25.txt) as it is the most comprehensive publicly available source of SV/CNV, and all variants in the database align with GRCh38. We used a reciprocal overlap of 70/90/95%.

## Supplementary Material

Supplement 1

## Figures and Tables

**Table 1:** Number of variants by SV type

Deletions	50740
Duplications	13000
Insertions	2684
Inversions	1359
Translocations	12

**Table 2:** Overview of frequency

Range	Count

(0–1%]	41863
(1–2%]	5114
(2–5%]	5770
(5–10%]	3812
(10–20%]	3507
(20–30%]	1857
(30–40%]	1294
(40–50%]	987
(50–60%]	820
(60–70%]	666
(70–80%]	619
(80–90%]	545
(90–100%]	941

**Table 3:** Count by range

Range	Count

(0–250]	30366
(250–500]	9012
(500–1000]	7966
(1000–2000]	6811
(2000–5000]	8761
(5000–10000]	4879

**Table 4:** Number of SVs by ACMG predicted impact

	Count
ACMG	

Benign	17078
Uncertain	46509
Likely pathogenic	138
Pathogenic	15

**Table 5:** Genes with predicted pathogenic variants. In some cases there are two variants in a gene, which are listed in separate rows. In some cases, a variants overlaps two genes, listed in the same row.

Chrom	Location	Length	SV Type	Genes	Disease	Significance

1	44837779	4532	DEL	*PTCH2*	?Nevoid Basal cell carcinoma syndrome	Disputed[24, 25]
5	78813976	2071	DEL	*ARSB*	Mucopolysaccharidosis type VI	Known population variant
6	64386321	5678	DUP	*EYS*	Retinitis Pigmentosa	Novel
6	109687391	6364	DEL	*AK9;FIG4*	Spermatogenic failure	Novel
10	26171085	5164	DEL	*MYO3A*	Deafness	Known African population variant
11	5226163	1392	DEL	*HBB*	B-Thalassemia	Known African population variant
11	5226626	7464	DEL	*HBB;HBD*	B-Thalassemia;Delta-Thalassemia	Known population variant
12	102865716	5349	DEL	*PAH*	Phenylketonuria	Known African population variant
14	74298906	4595	DEL	*ABCD4*	Methylmalonic aciduria and homocystinuria	Known African population variant
15	27975441	8930	DUP	*OCA2*	Albinism	Novel
15	28017719	2958	DEL	*OCA2*	Albinism	Known African population variant
16	173579	3773	DEL	*HBA1;HBA2*	Alpha-Thalassemia	Known African population variant
16	1356771	8145	DEL	*GNPTG;UNKL*	Mucolipidosis III gamma; N/A	Novel
18	62152637	5065	DEL	*PIGN*	Multiple congenital anomalies-hypotonia- seizures syndrome 1	Known African population variant
19	41417846	5857	DEL	*BCKDHA*	Maple Syrup urine disease	Novel

**Table 6:** Genomic location of variants

	Count
SV Category	

coding	3085
full gene	60
intergenic	36981
intronic	29105

**Table 7:** Sources of high-coverage WGS data for this study on 1,091 participants from multiple African populations. Depth=median depth of mapped sections of chromosomes 1–22. Data access details are given in the Data availability section.

Countries	Data Source	*n*	Depth

H3Africa — 446 samples			
Benin	University of Montréal	50	37
Burkina Faso, Ghana	AWI-Gen	60	37
Botswana	CAfGEN	47	37
Cameroon	University of Dschang	26	35
Cameroon	IFGeneRA	24	36
Mali	Clinical and Genetic Studies of Hereditary Neurological Disorders in Mali	50	31
Nigeria	Institute of Human Virology	49	36
Zambia	University of Zambia	41	36
South Africa	AWI-Gen	100	37

Other African collaborators: High coverage – 100 samples			

South Africa	SA Human Genome Programme	15	
South Africa	Cell Biology Research Lab, NICD/Wits	85	40

Public data sets – 545 samples			

Various	Simons Foundation	31	37
	
Gambia	1000 Genomes	113	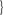 35
Kenya		99	
Nigeria		217	
Sierra Leone		85	

**Total**		**1091**	

## Data Availability

The results of our analysis are available at https://github.com/shaze/h3acnvcalls: BED files are available identifying all SV regions from all the analyses shown in Figure 1. All data are described in more detail in the Supplementary Material. The 1000 Genomes data and the SGDP data are publicly available. The other datasets used in this study are available from the European Genome-Phenome Archive (https://ega-archive.org/) on application to the relevant Data Access Committees: H3Africa project — EGAD00001006418 (South Africa), EGAD00001004220 (ACCME/Berom), EGAD00001004316 & EGAD00001004393 (Cameroon), EGAD00001004334 (Mali), EGAD00001004448 (AWI-Gen West Africa), EGAD00001004505 (Botswana), EGAD00001004557 (Benin); EGAD00001004220 (Zambia); Wits — EGAD00001007589 (South Africa); SAHGP — EGAD00001003791 (South Africa).
