# Supplementary material for: An assessment of the genomic structural variation landscape in Sub-Saharan African populations
Source: Res Sq. 2024 Jul 8:rs.3.rs-4485126. Preprint. [Version 1] doi: 10.21203/rs.3.rs-4485126/v1 (PMC11261963; doi:10.21203/rs.3.rs-4485126/v1)
Supplement: Supplement 1 [file NIHPPrs4485126v1-supplement-1.pdf]

Appendix A Supplementary methods section

Data set details

Cell Biology Research Laboratory HIV Study The Cell Biology Research Laboratory HIV study included 40 participants living with HIV-1, all of whom were Bantu-speaking South Africans except for one participant who was of mixed ancestry. Samples were prepared using the TruSeq Nano protocol (PCR-based). High coverage sequencing ($\approx 30\times$ was performed using the Illumina SeqLab workflow system and the Illumina HiSeqX platform. EGAD00001007589.

H3Africa-Baylor The H3Africa data set comprised 347 individuals from several H3Africa studies. Participants were from

- Benin, generated by the Awadalla, Lab, University of Montréal (EGAD00001004557),
- Botswana, generated by the Collaborative African Genomics Network – CAfGEN – (EGAD00001004505),
- Burkina Faso and Ghana, generated by the Africa Wits-INDEPTH Partnership for Genomic Research – AWI-Gen – (EGAD00001006418);
- Cameroon
  - 24 generated by the *Individual Findings in Genetic Research in Africa* project (IFGeneRA, EGAD00001004316)
  - 26 generated by the TrypanoGEN Project (EGAD00001004393).
- Mali, generated by the Clinical and Genetic Studies of Hereditary Neurological Disorders in Mali Project (EGAD00001004334)
- Nigeria generated by the African Collaborative Centre for Microbiome and Genomics Research – ACCME – (EGAD00001004220);
- Zambia, generated by TrypanoGEN (EGAD00001004220).

(South Africa), (ACCME/Berom), EGAD00001004316 & EGAD00001004393 (Cameroon), EGAD00001004334 (Mali), EGAD00001004448 (AWI-Gen West Africa), EGAD00001004505 (Botswana), EGAD00001004557 (Benin); EGAD00001004220 (Zambia); Wits — (South Africa); SAHGP — EGAD00001003791 (South Africa).
Samples were prepared using the TruSeq Nano DNA Library Prep Kits (PCR-based) and underwent WGS on an Illumina TenX (150 bp) to a minimum depth of coverage of 30x [13].

African Wits-INDEPTH Partnership for the Genomic Reseearch (AWI-Gen) The AWI-Gen study is a longitudinal study of over 10,000 participants investigating the interplay between genetic and environmental risk factors in cardiometabolic diseases [39]. The samples from Burkina Faso described above come from this project. The EGAD00001007589 data set comprises WGS from 100 of these randomly selected healthy individuals who were all South-Eastern Bantu speakers living in South Africa, and were sequenced by a different sequencing centre. Samples were prepared using a PCR-free method and sequenced on an Illumina HiSeq X Ten instrument with a minimum read depth of coverage of 30x.

Southern African Human Genome Programme EGAD00001003791 contains 15 samples from Bantu-speaking individuals. Samples were prepared using PCR-free methods for sequencing on the Illumina HiSeq 2000 instrument with a minimum read depth of coverage of 30x [30].

Simons Genome Diversity Project Twenty-five samples were selected from the 300 SSA individuals in the Simons Genome Diversity Project. These individuals were from Botswana, Congo, Kenya, Namibia, Nigeria, Senegal, South Africa, Sudan. Samples were sequenced at an average depth of 43x at Illumina Ltd.; almost all samples were prepared using the same PCR-free library preparation [29].

1000 Genomes Project Consortium A total of 500 African samples were included from the expanded 1000 Genomes Project where 3 202 samples were sequenced with deep coverage. Participants were Gambian Mandinka, Mende from Sierra Leone, Yoruba from Ibadan, Nigeria, Esan from Nigeria and Luhya from Webuye, Kenya. Samples were prepared using Truseq DNA PCR-free (450bp) Library Preparation Kit and sequenced on an Illumina Novaseq 6000 sequencer to an average depth of 30x [40]).

Genetic diversity

The genetic diversity of the African samples, and the importance of including African samples in genomic studies is shown in the principal component charts shown in Figure A1, which is based on single nucleotide variation. Using the samples of our study as a core dataset, to show context we randomly picked high-coverage samples from various 1000 Genomes Project populations – 115 European samples (randomly chosen from CEU, GBR and TSI); 87 South Asian samples (BEB); 41 East Asian samples (CHS and CHB); 158 samples of individuals in North America and the Caribbean of African descent (ASW and ACB); and 85 individuals from South America (PEL). All BAM files were aligned to GRCh38. We called variants individually using Deepvariant, and jointly called using GLnexus [41]. We then used PLINK [42] to LD prune and perform PCA.

A.1 Merging approach

Appendix B Supplementary results

B.1 Genes with likely pathogenic variants

The list of genes with likely pathogenic variants can be found in Table B1.

Table B1: List of genes that AnnotSV predicts to be likely pathogenic

Chrom	Length	Gene	Chrom	Length	Gene
1	8686	*MASP2*	10	4007	*ASCC1*

Continued on next page

Chrom	Length	Gene	Chrom	Length	Gene
1	9816	*CLCNKB;FAM131C*	10	723	*RGR*
1	2419	*AK2*	10	3331	*NDUFB8*
1	8573	*C8B*	10	722	*RBM20*
1	7819	*ALG14;ALG14-AS1*	11	344	*TALDO1*
1	4836	*DPYD*	11	7511	*CARS1*
1	3927	*SLC25A24*	11	4986	*HBG1;HBG2*
1	1003	*FLG*	11	4929	*HBG1;HBG2*
1	915	*FLG*	11	192	*PTH*
1	4605	*REN*	11	3087	*PTPRJ*
1	744	*LYST*	11	4994	*GPR137;KCNK4;KCNK4-TEX40*
1	1731	*FMN2*	11	2998	*TMEM126B*
2	1328	*LRPPRC*	11	2277	*TMEM126B*
2	5444	*PPP1R21*	11	2764	*TMEM126B*
2	5442	*PPP1R21*	11	268	*TMEM126B*
2	3315	*PPP1R21*	11	1195	*CTSC*
2	7323	*PPP1R21*	11	1274	*NECTIN1*
2	120	*CACNB4*	12	90	*PEX5*
2	9350	*CHROMR;PRKRA*	12	8617	*GDF3*
2	5291	*CHROMR;PRKRA*	12	4708	*PDE3A*
2	6850	PRKRA	12	4707	*ABCC9*
2	549	*PRKRA*	12	6269	*RXYLT1*
2	839	*MFF*	12	3672	*GNPTAB*
2	7703	*MFF*	12	983	*GNPTAB*
2	3270	*CHRND*	12	2437	*GNPTAB*
2	1226	*NDUFA10*	13	3726	*RCBTB1*
3	8189	*THRB*	13	2528	*RCBTB1*
3	249	*UQCRC1*	13	331	*CARS2*
3	6442	*IQCB1*	14	5075	*NUBPL*
3	4256	*TCTEX1D2*	14	5366	*COQ6;ENTPD5*
4	8339	*SLC2A9*	14	71	*IRF2BPL*
5	551	*SLC9A3;SLC9A3-AS1*	15	2708	*MYEF2;SLC24A5*
5	354	*SLC9A3*	15	9050	*LIPC;LIPC-AS1*
5	727	*TARS1*	15	3194	*PPIB;SNX22*
5	90	*MATR3*	15	697	*PPIB*
5	134	*ADAMTS2*	15	1294	*ACAN*
6	2116	*MAK*	15	6201	*ADAMTS17*
6	3307	*TNXB*	15	712	*CERS3*
6	1786	*TAPBP*	15	2518	*LRRK1*
6	6718	*EPM2A*	16	3621	*RPL3L*
6	3711	*SYNE1*	16	3512	*BICDL2;THOC6*
7	5831	*DNAH11*	16	5738	*ZP2*
7	369	*PKD1L1*	16	4246	*FCSK*
7	1222	*POR*	17	642	*MYO1C*
8	909	*RP1L1*	17	1443	*PFN1*
8	73	*RP1L1*	17	450	*SLC52A1*
8	7033	*MCM4*	17	5839	*SLC13A5*
8	357	*RAD21*	17	5775	*DNAH9*
8	7636	*RNF139*	17	9607	*MPO*
9	1205	*APTX*	17	297	*QRICH2*
9	5750	*SECISBP2*	17	119	*CCDC40*
9	2618	*SECISBP2*	17	228	*CARD14*

Continued on next page

Chrom	Length	Gene	Chrom	Length	Gene
9	3725	*SECISBP2*	17	264	*FSCN2*
9	5398	*SECISBP2*	17	121	*WDR45B*
9	2417	*SECISBP2*	17	1262	*TBCD*
9	7480	*SECISBP2*	19	1459	*GNA11*
9	6558	*SECISBP2*	19	4647	*C19orf12*
9	7632	*SPTLC1*	19	6158	*LIG1*
9	2251	*SPTLC1*	19	9237	*TRPM4*
9	1532	*CDK5RAP2*	20	73	*KIZ*
9	396	*CEL*	20	9699	*IFT52*
9	2215	*AGPAT2*	20	3337	*VAPB*
10	1712	*DHTKD1*	21	966	*CFAP298*;*CFAP298-TCP10L*
10	526	*ANKRD26*	21	52	*IFNAR1*
10	64	*PCDH15*	21	1240	*RSPH1*
10	5359	*DNA2*	21	1837	*TSPEAR*
10	1547	*DNA2*	22	494	*LZTR1*
10	2558	*KIFBP*	22	6084	*DMC1*
10	2684	*ASCC1*	X	314	*DNASE1L1;RPL10*

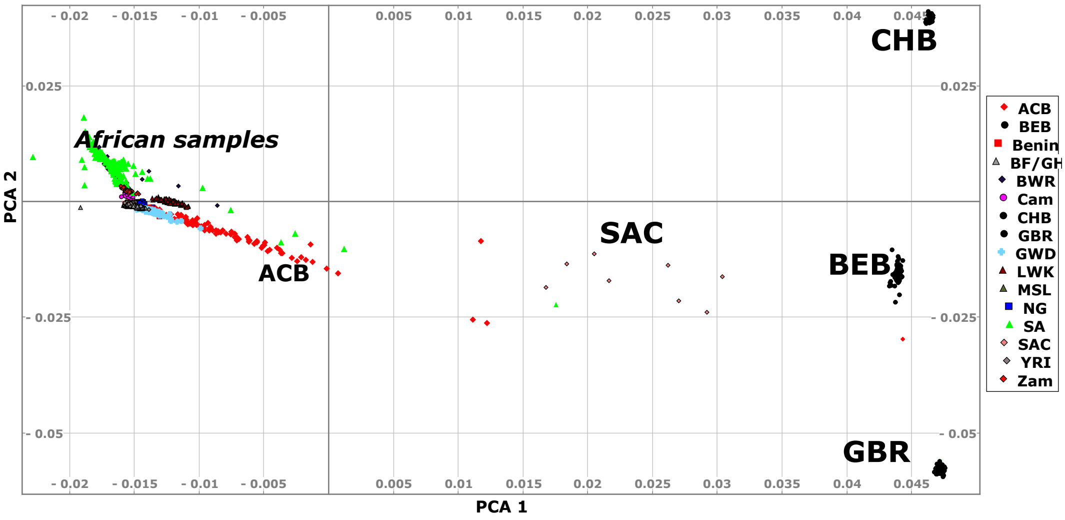

(a) Global Population PCA – the *African samples* were used in our study. The others show context

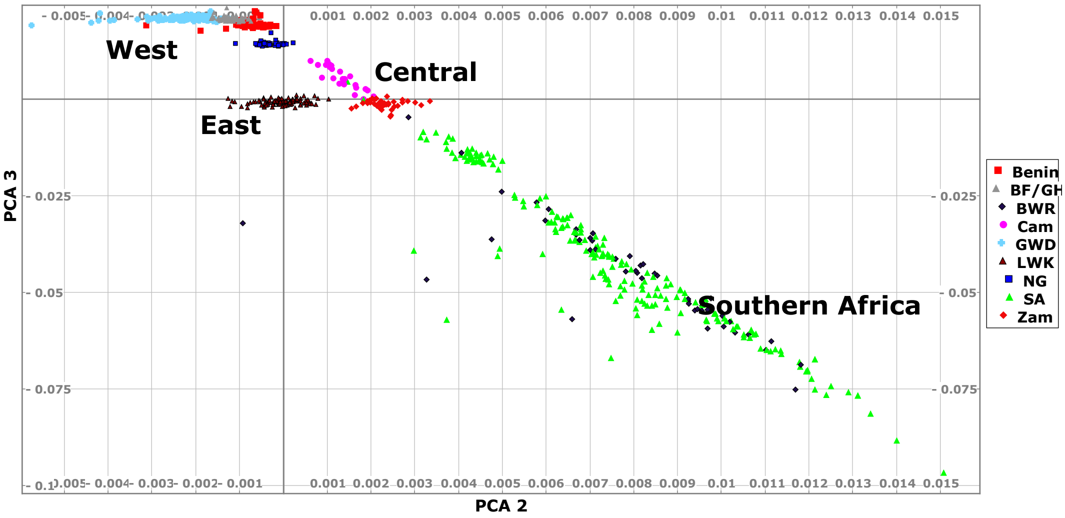

(b) Focus on our samples, with geographical regions. Some populations and outliers were removed for clarity.

Fig. A1: PCA of samples. Key: ACB – 1000G African Caribbean in Barbados; BEB – 1000G Bengali in Bangladesh; Benin – H3Africa; BF/GH – H3Africa Burkina Faso and Ghana; BWR – H3Africa Botswana; CAM – H3Afica Cameroon; CHB – 1000G Han Chinese; GBR – 1000G Great Britain; GWD – 1000G Gambian Western Districts; LWK – 1000H Luhya from Kenya; MSL – 1000G Mandinka Sierra Leone; NG – H3Africa Berom from Nigeria; SA – CBRL, H3Africa, Bantu-speakers from SAHGP South Africa; SAC – SA Coloureds in SAHGP; YRI —- 1000G Yoruba; Zam – H3Africa Zambians.
